# The Secrecy of the Proceedings at "Committees."

**Published:** 1900-02-24

**Authors:** 


### The Institutional Workshop.

### THE SECRECY OF THE PROCEEDINGS AT "COMMITTEES."

A very absurd position of affairs has come to light at Belper. The representatives of the Belper Urban District Council upon the Belper Isolation Hospital Committee have got across with their colleagues, and have resigned. We do not now propose to enter into the questions of hospital discipline which have led to such differences among the members of this committee. What we wish to draw attention to is the assumption, which we think is a totally wrong assumption, that the proceedings of such a committee as that which is constituted by law for the management of an isolation hospital are to be considered as confidential.

At the meeting of the District Council the public can attend, and reporters can be present, but the proceedings of committee are to be regarded as private. In explaining his reasons for resigning Mr. Strutt, who is chairman of the Belper Urban District Council, said that he felt in rather a difficult position, because what had taken place was done in committee. "This was," he said, "of course, private, but at the same time he thought some explanation was due from him to the Council," and then ensued a long discussion in which nothing was stated openly, all was about certain things which had been done, certain votes which had been taken, 'certain events which had happened, but no definite statement was made as to what things, what votes, &c., they were talking about. All because the proceedings at a committee are confidential. Now this seems to us to be altogether wrong. When a District or other Council appoints a committee to carry out certain specific duties the proceedings of that committee are confidential, but that is because the report is given in to the Council, and the Council as a whole is in the end responsible to those by whom it is elected for all its doings, and even for the doings of each of its committees.

But the Hospital Committee by which an isolation hospital is managed may consist of a number of members, appointed by the several Councils which together form the hospital district. Its powers are defined by Act of Parliament, and its proceedings cannot be revised or annulled by any one of the various Councils whose representatives sit upon it.

The secrecy which shields the proceedings of a committee of a Board or Council is proper enough, seeing that the whole Board or Council is responsible for every one of its acts; but the proceedings of a Hospital Committee, which is appointed by several Councils, and for the acts of which no individual Council is responsible, should undoubtedly be open to the fullest publicity, otherwise they are absolutely relieved from criticism and from the pressure of public opinion—which perhaps accounts for some of their vagaries. The suggestion that the proceedings of such a body are confidential is a mere bogie, arising from the name "committee," a name for which those who drafted the Isolation Hospitals Act are alone responsible, and one which is in the Act entirely divorced from its ordinary meaning. If this body had been called a Hospital Board no one would ever have thought of its proceedings any more than those of the Metropolitan Asylums Board being held to be confidential.

